# Cyclosporine A does not prevent second-eye involvement in Leber’s hereditary optic neuropathy

**DOI:** 10.1186/s13023-018-0773-y

**Published:** 2018-02-17

**Authors:** Stéphanie Leruez, Christophe Verny, Dominique Bonneau, Vincent Procaccio, Guy Lenaers, Patrizia Amati-Bonneau, Pascal Reynier, Clarisse Scherer, Adriana Prundean, Christophe Orssaud, Xavier Zanlonghi, Marie-Bénédicte Rougier, Caroline Tilikete, Dan Miléa

**Affiliations:** 1 Service d’Ophtalmologie, CHU Angers, 49000 Angers, France; 2 Institut MITOVASC, UMR CNRS 6015-INSERM1083, Université d’Angers, 49000 Angers, France; 3 Centre de référence des maladies neurogénétiques, Service de Neurologie, CHU Angers, 49000 Angers, France; 4 Unité Fonctionnelle d’Ophtalmologie, Centre de référence des Maladies Rares en Ophtalmologie OPHTARA, Hôpital Européen Georges Pompidou, Assistance Publique Hôpitaux de Paris, 75015 Paris, France; 5 Centre de compétence maladie rare, Clinique Jules Verne, 44300 Nantes, France; 6 Service d’Ophtalmologie, CHU de Bordeaux, 33000 Bordeaux, France; 7 Unité de Neuro-Ophtalmologie, Hospices Civils de Lyon, Hôpital Neurologique, 69677 Bron, France; 8 Singapore Eye Research Institute, Singapore National Eye Centre and Duke-NUS, Singapore, Singapore; 9 Angers University Hospital, Angers, France

**Keywords:** Leber’s hereditary optic neuropathy, Second-eye involvement, Cyclosporine

## Abstract

**Backrground:**

Evaluation of the efficacy of oral cyclosporine A as a prophylactic agent in preventing second-eye involvement in Leber’s hereditary optic neuropathy (LHON) in a prospective, open-label, non-randomized, multicenter pilot study. Only LHON patients aged 18 years or more, with confirmed primary mitochondrial DNA mutations and strictly unilateral optic neuropathy occurring within 6 months prior to enrolment, were included in the study. All these patients, receiving treatment with oral cyclosporine (Neoral®, Novartis) at 2.5 mg/kg/day, were examined at three-month intervals for a year. The primary endpoint was the best corrected visual acuity in the unaffected eye; the secondary endpoints were the best corrected visual acuity in the first eye affected, the mean visual field defect on automated perimetry, the thickness of the perifoveal retinal ganglion cell inner plexiform layer, and the thickness of the peripapillary retinal nerve fiber layer in both eyes.

**Results:**

Among the 24 patients referred to our institution with genetically confirmed LHON, between July 2011 and April 2014, only five patients, four males and one female, fulfilled the inclusion criteria. Age at enrolment ranged from 19 to 42 years (mean: 27.2 years; median: 26 years), four patients harbored the m.11778G > A pathogenic variant, and one the m.14484 T > C pathogenic variant. The time-interval between the onset of symptoms and inclusion in the study ranged from 7 to 17 weeks (mean: 11.8 weeks; median: 9 weeks). Despite treatment with oral cyclosporine A, all patients eventually experienced bilateral eye involvement, occurring within 11–65 weeks after the initiation of treatment. Over the study time period, the average best corrected visual acuity worsened in the first eye affected; by the end of the study, both eyes were equally affected.

**Conclusions:**

Oral cyclosporine, at 2.5 mg/kg/day, did not prevent second-eye involvement in patients with strictly unilateral Leber’s hereditary optic neuropathy.

**Trial registration:**

ClinicalTrials.gov Identifier: NCT02176733. Registrated June 25, 2014.

## Background

Leber’s hereditary optic neuropathy (LHON, OMIM 535000), with a prevalence higher than 3/100,000, is the most common primary mitochondrial DNA (mtDNA) disorder. LHON is clinically characterized by an acute and painless visual loss occurring typically but not exclusively in young men [1], sequentially affecting both eyes within weeks or months [2]. The median delay of inter-eye involvement is 6–8 weeks [3], although retarded bilateralization has been reported [4]. Simultaneous bilateral involvement may occur in up to 25% of cases [3]. Visual field defects include central or centrocecal scotomas, associated with low vision, usually worse than 20/200. The pupillary light reflex is generally mildly affected in unilateral cases [5]. At the acute stage, fundoscopy typically reveals peripapillary telangiectatic microangiopathy and thickening of the peripapillary retinal nerve fiber layer (RNFL). As the disease progresses, atrophy of the optic disc becomes progressively visible. Three primary mtDNA mutations, at positions m.11778G > A, m.3460G > A and m.14484 T > C in the *MT-ND4*, *MT-ND1*, and *MT-ND6* genes, respectively, account for about 90% of all LHON cases. LHON, associated with the m.14484 T > C mutation, has the best visual prognosis, with spontaneous recovery occurring in up to 65% of cases [6].

Despite recent advances in the understanding of the pathophysiology of mitochondrial disorders, only a few randomized controlled treatment trials have been evaluated in LHON. Most of the treatments tested - including vitamins B, C, and E, folic acid, coenzyme Q10 [7], and other drugs expected to stimulate mitochondrial biogenesis - were not effective. In a randomized, placebo-controlled clinical trial, LHON patients received either 900 mg/day of idebenone or placebo [8]. Even though this trial failed to show any significant improvement of visual acuity after idebenone treatment, a post hoc interaction analysis indicated that patients with asymmetric inter-ocular visual acuities, thus with relatively recent symptoms of LHON, were more likely to benefit from this treatment [9]. Brimonidine, a topical a-2 agonist commonly used to lower intraocular pressure in glaucoma, has also been tested as a potential prophylactic agent to prevent second-eye involvement. In an open-label trial including nine patients with unilateral acute loss of vision secondary to LHON [10], brimonidine treatment did not prevent second-eye involvement and loss of vision. Among other therapeutic strategies, gene therapy, based on the intravitreal injection of an adeno-associated virus allotopically expressing an mtDNA-encoded gene, is also considered to hold promise for treating LHON [11]. However, gene therapy is restricted so far only to the affected eye [12].

Among several potential drugs that have been tested in vitro in cellular models of LHON, cyclosporine A appears to be an interesting candidate as a potent inhibitor of the opening of the mitochondrial permeability transition pore [13], which plays a crucial role in damage-induced cell death [14]. Indeed, cyclosporine A, which inhibits this channel through its binding to the peptidylprolyl isomerase, cyclophilin D, located in the mitochondrial matrix, may protect retinal ganglion cells (RGCs) from death [12]. The aim of our study was to investigate the clinical efficacy of the administration of low doses of oral cyclosporine A in preventing second-eye involvement in patients with strictly unilateral, genetically confirmed LHON.

## Methods

Thirteen French centers participated in this prospective, open-label, phase II, non-randomized, multicenter trial aimed at evaluating the efficacy and tolerance of low doses of oral cyclosporine A in patients with unilateral LHON, occurring within 6 months of onset. Prior informed written consent was obtained from all the patients participating in the trial. The study was conducted in accordance with the ethical standards set forth in the Helsinki Declaration (1983). The protocol was approved by the local Ethical Review Committee and by the French Health Products Safety Agency (n°2011–001214-34).

The criteria for inclusion were the following: male or female patients, at least 18 years old, with genetically confirmed LHON with an onset of less than 6 months, affecting strictly one eye. The criteria for exclusion were: age under 18 years, bilateral ophthalmic involvement, duration of symptoms beyond 6 months, other associated ophthalmic conditions, pregnancy, and lack of health insurance coverage. Molecular diagnosis of LHON was performed on blood samples from patients, using next-generation sequencing with an Ion Proton system (Thermo Fisher Scientific, Waltham, MA, USA). The protocols and primers used for mtDNA sequencing are available upon request. The heteroplasmy of the m.11778G > A and m.14484 T > C pathogenic variants was quantified as described elsewhere [15].

The demographic data, medical and ophthalmic histories of the patients were recorded at the initial visit. Patients were examined at day 0 (baseline), and at 3, 6, 9, and 12 months after inclusion in the study. At each visit, the best corrected visual acuity (BCVA) was determined in both eyes by using the Early Treatment Diabetic Retinopathy Study (ETDRS) method and the Parinaud visual acuity chart at 33 cm for near vision. The BCVA rating was then converted to logMar units. Standard automated perimetry with the Humphrey visual field analyzer (Carl Zeiss, Dublin, CA, USA), using the 24–2 SITA-fast algorithm, was performed at each visit, and the visual field data were analyzed in terms of the mean defect (MD) in decibels (dB). Other data collected included vital signs, results of slit-lamp microscopy, measurement of intraocular pressure, the appearance of the optic disk in fundoscopy (normal, pallor, telangiectasia, elevation or other abnormalities), color vision with Lanthony’s desaturated 15-hue test, and the Mars letter contrast sensitivity test. High-definition optical coherence tomography (HD-OCT) was performed at inclusion and at each follow-up visit, using the Cirrus device (software version 6.0, Carl Zeiss Meditec, Dublin, CA, USA) to measure the thickness of the perifoveal retinal ganglion cell-inner plexiform layer (GC-IPL) and of the peripapillary retinal nerve fiber layer (RNFL). Segmentation and measurement of GC-IPL were automatically executed with the ganglion cell analysis (GCA) developed for the Cirrus HD-OCT device. The GCA algorithm identifies the thickness of the GC-IPL layer, defined by the outer boundary of the RNFL and the outer boundary of the inner plexiform layer (IPL).

All the patients received oral doses of cyclosporine (Neoral®, Novartis, Rueil-Malmaison, France) at 2.5 mg/kg/day, which is in the lower range of doses used for immunosuppression [16]. The treatment was administered during 9 months after the initial inclusion or until the occurrence of second-eye involvement. The residual blood concentration of cyclosporine was measured 5 days after the initiation of treatment and at 1, 3, 6 and 9 months of treatment. The blood creatinine level was evaluated every 2 months from the third month of treatment.

The primary end-point was the BCVA of the unaffected eye at the end of the study. We have defined « second eye involvement » any significant deterioration of its visual acuity, i.e. worsening of + 0,1 logMar or more. The main secondary end-point was the change of the BCVA in the first-involved eye. Other secondary end-points were the following: the mean visual field defect, the thickness of the perifoveal retinal GC-IPL, and the thickness of the peripapillary RNFL. Statistical analyses were performed using the R software package. The level of statistical significance was set at *p* < 0.05. The comparison between the baseline data and the results of the exit examination, and the inter-eye comparison were performed using Student’s *t*-test for the BCVA, the mean visual field defect, the thickness of the GC-IPL and that of the RNFL, after applying the F-test for the equality of two variances.

## Results

### Baseline clinical data (Table 1)

Among the 24 LHON patients referred to our center between July 2011 and April 2014, only five patients, four men and one woman, met the inclusion criteria of a strictly monocular involvement within the time-frame considered. The age at enrolment ranged from 19 to 42 years (mean: 27.2 years, median: 26 years). Four patients harbored the m.11778G > A pathogenic mtDNA variant, and one patient harbored the m.14484 T > C variant, with a mutant load ranging from 97% to 100%. None of the patients had any extraocular features. At the end of the study, one patient had been followed up during 15 months, two patients during 12 months, and two patients during 6 months. Nineteen patients were excluded from the study for the following reasons: age under 18 years (*n* = 4), visual impairment lasting for more than 6 months (*n* = 2), visual impairment affecting both eyes (*n* = 9), contraindications for cyclosporine (*n* = 3), and lack of health insurance (*n* = 1).

**Table 1:** Clinical data of five LHON patients at baseline and exit examinations during the cyclosporine trial

Patient	Age	Gender	mtDNA mutation	Heteroplasmy level (%)	Baseline Visual function First eye		Baseline visual function Second eye		Time between initial visual loss and beginning of treatment (weeks)	Time between beginning of treatment and second eye visual loss (weeks)	Time between involvement of the two eyes (weeks)	Exit Visual Function First Eye		Exit Visual Function Second Eye	
					Visual acuity (logMar) [Snellen]	Visual field (MD)	Visual acuity (logMar) [Snellen]	Visual field (MD)				Visual acuity (logMar) [Snellen]	Visual field (MD)	Visual acuity (logMAr) [Snellen]	Visual field (MD)
1	42	F	11778	98.17	0.8 [20/125]	−1.24	0 [20/20]	0.65	16	15	31	2.1 [HM]	−32.55	2.1 [HM]	−32.08
2	30	M	11778	100	0.5 [20/63]	−2.89	0 [20/20]	−1.88	17	11	28	1.6 [20/800]	−22.85	1.1 [20/250]	−12.41
3	26	M	11778	98	1.1 [20/250]	−7.81	0 [20/20]	−2.17	9	65	74	2.1 [HM]	−33.99	2.1 [HM]	−29.49
4	19	M	14484	100	0.7 [20/100]	−4.97	−0.1 [20/16]	−1.45	6	12	18	0.9 [20/160]	−25.77	1 [20/200]	−29.28
5	19	M	11778	97	1.1 [20/250]	−8.86	−0.1 [20/16]	−2.11	9	18	27	2.40 [HM]	−34.62	1.2 [20/320]	−18.94
Mean	27.2	–	–	98.63	0.84 [20/125]	−5.15-	0 [20/20]	−1.392	11.4	24.2	35.6	1.82 [CF]	−29.96	1.50 [20/500]	−24.44
Median	26	–	–	98.17	0.8 [20/125]	−4.97	0 [20/20]	−1.88	9	15	28	2.1 [HM]	−31.25	1.2 [20/300]	−26.86

The time interval between the onset of visual loss and inclusion in the study ranged from 7 to 17 weeks (mean: 11.8 weeks, median: 9 weeks, standard deviation: (SD) 4.9 weeks). Three weeks after starting therapy with cyclosporine A, one of the patients (Patient 4) received an additional oral treatment of idebenone (Raxone®, Santhera, London, UK) at 300 mg three times a day.

**Fig. 1:**
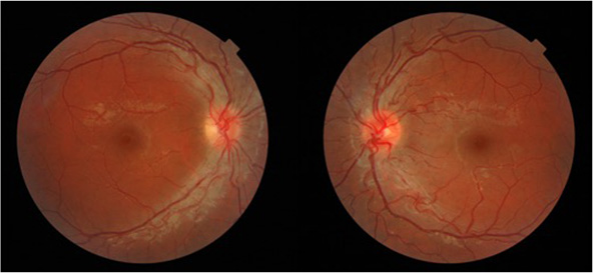
Fundoscopy of an 18-year-old patient with Leber’s hereditary optic neuropathy and recent visual loss (20/250) in his right eye, showing peripapillary telangiectatic microangiopathy in both eyes, despite normal visual function in the left eye

In the eyes first affected, the baseline BCVA ranged from + 0.5 to + 1.1 logMar (mean: + 0.84 logMar, median: + 0.80 logMar), which is equivalent to Snellen acuity values of 20/160 to 20/125, the mean visual field deviations ranged from − 1.24 dB to − 8.86 dB (mean: − 5.2 dB, median: − 4.97 dB, SD: 3.2 dB), the thickness of the RNFL ranged from 111 μm to 186 μm (mean: 141 μm, median: 141 μm, SD: 28 μm); and the thickness of the GC-IPL ranged from 66 μm to 79 μm (mean: 72.8 μm, median: 73 μm, SD: 5.8 μm). Baseline fundoscopy revealed abnormalities in all the first-involved eyes, including the presence of telangiectasic vessels in three cases (Fig. 1), and pseudo-papilledema in two cases.

In all the patients, the second eye was asymptomatic at initial assessment with a baseline BCVA ranging from + 0.2 logMar to − 0.1 logMar (mean: 0 logMar, median 0 logMar); the mean deviation (MD) of the visual field ranged from + 0.65 dB to − 2.17 dB (mean: − 1.4 dB, median: − 1,88 dB, SD: 1.2 dB); the average RNFL thickness ranged from 87 μm to 124 μm (mean: 112 μm, median: 119 μm, SD: 15 μm); and the average thickness of the GC-IPL ranged from 72 μm to 87 μm (mean: 80.4 μm, median: 82 μm, SD: 5.9 μm). However, baseline fundoscopy of the second eye revealed telangiectasia in two of the five patients (Fig. 1).

### Second-eye involvement

In all five patients, the initially unaffected eye became secondarily involved between 11 to 65 weeks (mean: 24 weeks, SD: 23 weeks) after the beginning of the study.

### Clinical data assessed at the end of the study (Table 1)

At the end of the follow-up period, the BCVA of the first eye affected ranged from worse than + 2 logMar to + 0.9 logMar, with a mean of + 1.82 logMar (median: + 2.1 logMar, SD: 0.48 logMar); the MD of the visual field ranged from − 22.85 dB to − 34.62 dB (mean: − 30.0 dB, median − 31.25 dB, SD: 5.3 dB); the average thickness of the RNFL ranged from 53 to 133 μm (mean: 97 μm, median: 93 μm, SD: 35 μm); and the average thickness of the GC-IPL decreased sharply, ranging from 49 to 55 μm (mean 51.3 μm, median: 50.5 μm, SD 2.9 μm).

The BCVA of the second eye ranged from less than + 2.1 logMar to + 1 logMar (mean: + 1.50 logMar, median: + 1.20 logMar, SD: 0.50 logMar); the mean visual field deviations ranged from − 12.41 dB to − 32.08 dB (mean: − 24.4 dB, median: − 26.86 dB, SD: 8.4 dB); the average thickness of the RNFL ranged from 79 to 143 μm (mean: 122 μm, median: 109 μm, SD: 37 μm); and the average thickness of the GC-IPL ranged from 49 to 57 μm (mean: 56.8 μm, median: 56.5 μm, SD: 6.5 μm). At the end of the study, fundoscopy revealed optic disk pallor in all patients.

### Comparison between assessments at the beginning and the end of the study (Table 2)

**Fig. 2:**
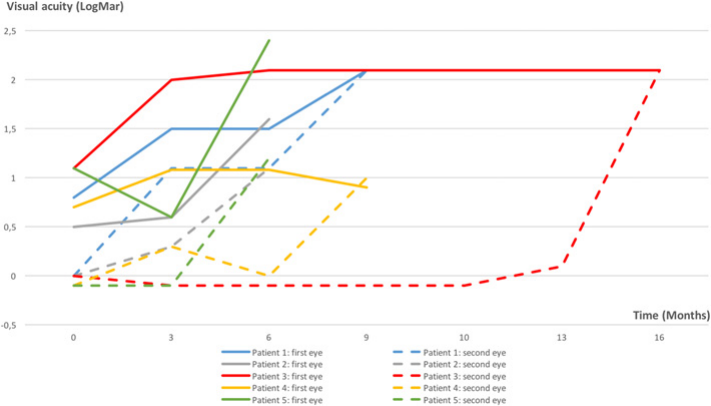
Graph displaying the visual acuities of included patients (full lines indicate the visual acuity in the first involved eye and dotted lines indicate visual acuity in the second involved eye. Blue lines: patient 1; grey lines: patient 2; red lines: patient 3; yellow lines: patient 4; green lines: patient 5

In all five patients, the visual acuity deteriorated significantly during the study, both in the first affected eye (*p* = 0.009) and in the second affected eye (*p* = 0.001) (Fig. 2). During the follow-up period, visual field defects worsened significantly in the first affected eye (*p* < 0.001) and in the second affected eye (*p* = 0.004). There was also a decrease in the average thickness of the GC-IPL in the first affected eye (*p* < 0.001) and in the second affected eye (*p* < 0.01), but there was no significant difference in the average thickness of the RNFL.

**Table 2:** Evolution of the first eye affected and the second eye in five LHON patients during the cyclosporine trial

		First Eye			Second Eye			Inter eye comparaison at exit
		Mean values at baseline	Mean values at exit	*P- value*	Mean values at baseline	Mean values at exit	*P- value*	*P- values*
Visual acuity (logMar)		0,84	1,82	0,009*	0	1,50	0,001*	0,40
OCT	Average RNFL thickness (μM)	142	97	0,057	112	122	0,57	0,30
	Average GC-PL thickness (μM)	72,8	51,9	< 0,001*	80,4	56,8	< 0,001*	0,18
Visual visual field defect (dB)		−5,2	−30,0	< 0,001*	−1,4	−24,4	0,004*	0,25

*P*-values calculated with Student’s *t-test*, *Significance of the bold datas

### Inter-eye comparison (Table 2)

At the end of the study, there was no significant inter-ocular difference in the BCVA (*p* = 0.40), the MD (*p* = 0.25), the average thickness of the RNFL (*p* = 0.30), or the average thickness of the GC-IPL (*p* = 0.18).

### Safety and tolerance of cyclosporine administration

All the LHON patients were evaluated during the study for the safety and tolerance of cyclosporine. Doses were adjusted according to blood concentrations (normal 100-300 μg/L) in 3 individuals. Minor side effects were reported by four patients: nausea (*n* = 1), diarrhea (*n* = 2), dizziness (*n* = 1), tremor (*n* = 2), hot/cold hands or feet (*n* = 3), otitis media (*n* = 2), headache (*n* = 1), angina (*n* = 1), psoriasis (*n* = 1), and the influenza syndrome (*n* = 1). One patient (patient 3) experienced a serious adverse event consisting of a high blood creatinine level after 2 months of cyclosporine treatment, without any other clinical or other biochemical abnormalities. Recovery of the renal function occurred 1 month after cyclosporine was stopped. Patient 2 dropped out from the study at 7.5 months, with no further follow-up.

## Discussion

In our study, the treatment of LHON patients suffering from a recent monocular loss of vision with low doses of orally administered cyclosporine failed to prevent second-eye involvement. Thus, the primary endpoint of the study - preservation of visual acuity in the second eye- was not achieved. Despite cyclosporine treatment, second-eye involvement occurred in all five patients included in the study, resulting in severe loss of vision, down to 20/200 or less. In addition, there was also a worsening of the visual acuity, the mean visual field defect, and the average thickness of the GC-IPL in the first eye affected. Concerning the secondary endpoints of the study, no significant differences were found between the visual functions of the two eyes in terms of visual acuity, the mean visual field defect, the average thickness of the RNFL, or the average thickness of the GC-IPL.

The demographic and clinical features of our group of LHON patients were comparable to those previously reported in the literature, with male predominance and the onset of visual loss occurring during the second and third decade of life, except for one patient who became symptomatic at age 42 years. The severity of visual loss in the first eye at baseline and in both eyes at the end of the study was worse than 20/200 (+ 1 logMar), which is classical at the nadir of the disease [3]. Similarly, the visual field of the first eye affected in all the patients showed central and centrocecal defects typical of LHON. The fundoscopic appearance of the first eye affected included telangiectatic vessels, optic disk hyperemia, and elevation or “swelling” in all five patients. The m.11778G > A mutation, one of the most common pathogenic mtDNA variants, responsible for about 70% of all LHON cases worldwide, was found in four of our five patients. The patients were homoplasmic, or almost homoplasmic, for a primary mtDNA mutation, as is the case for the majority of individuals affected with LHON [3, 17].

**Fig. 3:**
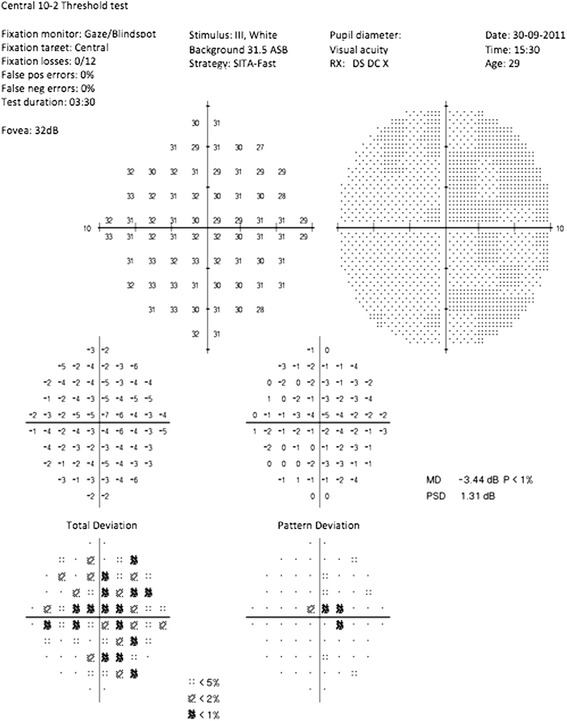
Visual fields in a patient with an asymptomatic eye at the initial visit. Despite normal visual acuity (20/20), there is a relative central depression on the pattern deviation plot

Our study included only the LHON patients with a strictly unilateral optic neuropathy, the second eye having a normal visual function. At baseline, the visual acuity of the second eye was 0 logMar, or better, in all patients; however, four of the five patients had minor visual field defects at some points of the central automated perimetry (Fig. 3). Some studies have reported central abnormalities of the visual field in asymptomatic carriers of LHON mtDNA mutations, e.g. maternal relatives of patients, but it remains unclear whether this is a benign marker of the disease or an early sign preceding loss of vision. These subtle abnormalities suggest that the apoptotic process might have already started in the supposedly unaffected eye at the time of enrolment, which may partly explain the failure of cyclosporine in preventing second-eye involvement.

Sequential optic nerve involvement is common in LHON, with about 75% of patients having an interval of 6–8 weeks between the onset of visual impairment of both eyes [3]. More than 97% of LHON patients develop second-eye involvement within 1 year, with a median delay of 6–8 weeks [18]. The median interval of 28 weeks of inter-ocular involvement in our series is longer than the average interval reported in the literature [19]. The time interval between the initiation of treatment and visual loss affecting the second eye ranged between 11 and 65 weeks, but the non-comparative design of our study does not allow us to conclude that cyclosporine delayed progression of the disease.

**Fig. 4:**
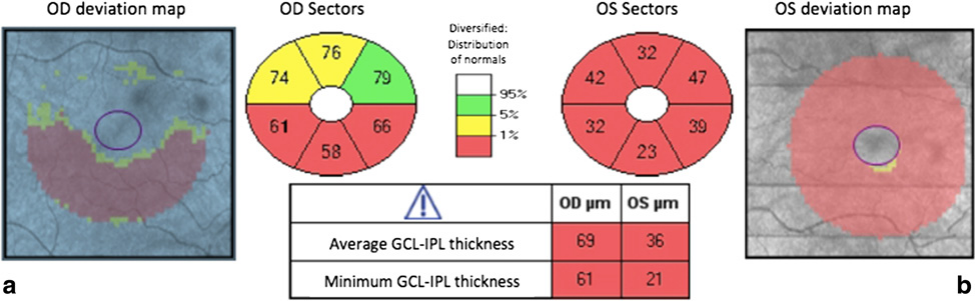
Optical coherence tomography, GC-IPL thickness map. **a** SD-OCT showing the GC-IPL thickness map in the right unaffected eye at baseline examination; in the inferior sector, there is a thinning of the GC-IPL before the acute stage of the disease. **b** The thinning of the GC-IPL already involves a full quadrant in the affected left eye

The sequential RNFL changes in our patients were comparable to those previously reported [20, 21]. Indeed, our findings showed that the RNFL was thicker in the second eye compared to baseline values, mainly in the inferior and superior quadrants before second-eye involvement. The thinning of the GC-IPL in the second eye was detectable before the symptomatic stage, affecting the inner ring of the nasal sector before expanding progressively in a centrifugal manner [22]. One patient displayed a different pattern of GC-IPL thinning in the inferior sector, occurring prior to the acute stage (Fig. 4).

Compared to the LHON patients carrying the m.11778G > A mutation, the patient harboring the m.14484 T > C variant had a better outcome. Six months after the end of the study, his visual acuity improved to 0 logMar in the first eye affected and to − 0.1 logMar in the second eye. However, the mean visual field defect did not improve in either the first eye affected or the second eye, remaining at -26 dB and -19 dB, respectively. The improved visual acuity in this case might represent a case of spontaneous recovery associated with the m.14484 T > C variant in contrast to the m.11778G > A mutation. It is also possible that this patient may have adapted to a new retinal re-fixation site, away from the central scotoma. In addition, since this patient had received an additional oral treatment of idebenone at 900 mg/day 3 weeks after starting on cyclosporine, the two treatments might have had synergistic effects.

Several explanations may be put forward for the failure of cyclosporine in preventing second-eye involvement in the LHON patients in our trial. First, oral cyclosporine may simply not be an appropriate treatment, at least at the regimen used. Alternatively, the dose of cyclosporine administered, based on that successfully used in individuals affected with collagen VI myopathy [16], may have been insufficient. Finally, cyclosporine treatment may have been initiated too late after the pathological process had set in. Indeed, four of the five patients studied had shown subtle abnormalities of the central visual field of the second eye at presentation (Fig. 3), suggesting that the window for prophylactic treatment might be narrower than previously thought [10].

## Conclusion

In our trial, oral low-dose cyclosporine failed to prevent involvement of the second eye in LHON, at least at the regimen used. Thus, other therapeutic options will have to be considered in the future to prevent second-eye involvement in this devastating condition.

## Abbreviations

BCVA: Best corrected visual acuity
dB: Decibels
ETDRS: Early Treatment Diabetic Retinopathy Study
GCA: Ganglion cell analysis
GC-IPL: Ganglion cell-inner plexiform layer
HD-OCT: High-definition optical coherence tomography
IPL: Inner plexiform layer
LHON: Leber’s hereditary optic neuropathy
MD: Mean defect
mtDNA: Mitochondrial DNA
RGCs: Retinal ganglion cells
RNFL: Retinal nerve fiber layer
SD: Standard deviation
